# Evaluation of one-piece zirconia dental implants: An 8-year follow-up study

**DOI:** 10.1007/s00784-023-04935-1

**Published:** 2023-06-05

**Authors:** Stella Kiechle, Anja Liebermann, Gerson Mast, Marius Heitzer, Stephan Christian Möhlhenrich, Frank Hölzle, Heinz Kniha, Kristian Kniha

**Affiliations:** 1 Department of Oral and Cranio-Maxillofacial Surgery, University Hospital, Ludwig Maximilian University of Munich, Munich, Germany; 2 Department of Prosthetic Dentistry, University of Cologne, Faculty of Medicine and University Hospital Cologne, Kerpener Strasse 32, 50931 Cologne, Germany; 3 Department of Oral and Cranio-Maxillofacial Surgery, University Hospital RWTH Aachen, Pauwelstraße 30, Aachen, Germany; 4 Department of Orthodontics, University of Witten/Herdecke, Alfred-Herrhausen Str. 45, Witten, Germany; 5 Private Clinic for Oral Surgery, Dres. Kniha, Rosental 6, Munich, Germany

**Keywords:** Zirconia, Ceramic, Dental implant, Long term, Bone crest

## Abstract

**Objectives:**

Long-term studies of modern zirconia implants are still insufficient. This prospective 8-year follow-up study investigated one-piece zirconia implants.

**Materials and methods:**

Patients who had received a one-piece zirconia dental implant (PURE ceramic implant, Institut Straumann GmbH, Basel, Switzerland) were included in this study. Next to the implant survival and success rates, the radiographic and clinical implant parameters were assessed.

**Results:**

The overall survival rate of 67 zirconia implants in 39 patients was 100%. The overall success rate was 89.6%. Around the immediate zirconia implants, the success rate was 94.7%, and around the delayed implants, 87.5%. The immediate implants showed a significantly higher bone crest compared to the delayed implants (*p* = 0.0120). According to the pink esthetic score, the immediate implants revealed more favorable esthetic results compared to the delayed implants after an 8-year follow-up (*p* = 0.0002).

**Conclusions:**

After 8 years, the one-piece zirconia implants presented an 89.6% success rate. Regarding the timing of implantation, in individual cases, immediate implantation can have slight advantages over delayed implantation.

**Clinical relevance:**

Immediate implants can also be considered for zirconia implants and should not be excluded on principle.

## Introduction

Titanium implants osseointegrate into the human jaw and can thus support dentures. Provided there are no relevant interferences, this connection is permanent. As an implant material, titanium is the gold standard based on numerous long-term studies [1]. Titanium proved to be a biologically suitable material on which chemical bonds can form with the surrounding tissues, which are also sufficiently stable biomechanically [2]. When it comes to replacing missing teeth, they offer a therapeutic option that has become an indispensable part of modern dentistry [3].

However, titanium is not necessarily without any disadvantages. If the soft-tissue situation is unfavorable, especially in the region of the anterior teeth and anterior premolars with a high smile line, the gray color of the titanium may shine through the tissue as a complication [4, 5].

At present, titanium is predominantly used in implantology, but several studies have shown that titanium and zirconia are at least equivalent functionally, such as with regard to their osseointegration [6–9]. Due to its excellent biomechanical properties, zirconia has clearly prevailed over other ceramic materials, such as aluminum hydroxide ceramics [10–12]. Furthermore, zirconia implants have already achieved clinical results in various short-term studies. In a meta-analysis, after a year of observation, the all-ceramic reconstructions supported by ceramic implants showed good survival rates [13]. Additionally, in 39 patients, between 3-month and 3-year follow-ups, the papilla height significantly improved in the interdental space [14]. Furthermore, in terms of the total number of bacterial cells and the *T. forsythia* and *P. intermedia* bacterial values, the soft tissues around the zirconia implants had a lower inflammatory response to the experimental plaque formation than those around the titanium implants [15]. However, there is a lack of long-term studies on the often critical positioning toward zirconia implants.

The primary aim of the present prospective 8-year follow-up study was to evaluate the survival and success rates of immediate and delayed zirconia implants. Furthermore, clinical and radiographic images were analyzed for the hard- and soft-tissue parameters.

## Methods

A total of 39 patients and 67 zirconia implants (one-piece zirconia dental implant, PURE ceramic implant, Institut Straumann GmbH, Basel, Switzerland) were included in the present study. Zirconia monotype implants, each with a diameter of 4.1 mm, were used. The implants were available in lengths of 8, 10, and 12 mm and in two different abutment heights of 4.0 mm and 5.5 mm. A transmucosal implant placement was performed in all cases.

The patient population was related to that in a previously published investigation [16]. Of the original 87 patients, 39 patients (with a total of 67 implants) agreed to participate in this follow-up study, which was from March 2020 to July 2022. All 39 patients were available in the clinic within the indicated time period and were evaluated according to the indicated criteria without further dropouts. The ethics committee of the local medical faculty university reviewed and approved the study protocol (No. 20-040). The study was conducted in accordance with the principles of the Declaration of Helsinki. As the study was a prospective observational one, it was conducted in accordance with the STROBE (Strengthening the Reporting of Observational Studies in Epidemiology) statement [17].

### Experiment protocol

**Fig. 1:**
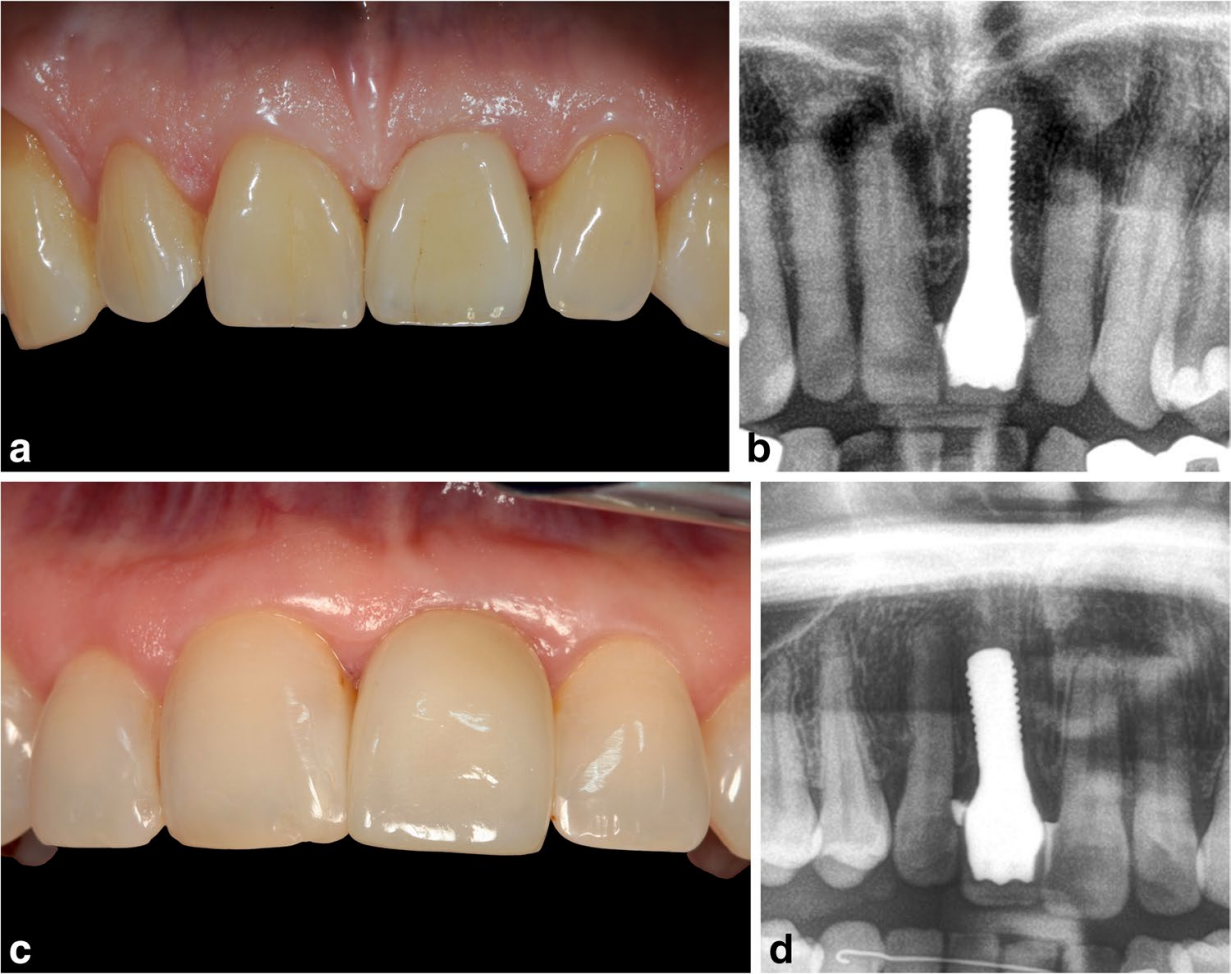
**A** Clinical follow-up image and **B** radiographic control of an immediate zirconia implant after 8 years. **C** Clinical follow-up image and **D** radiographic control of a delayed-implant case

The primary outcome variables were the implant survival and success rates, with a comparison of those of immediate and delayed implants (Fig. 1). If the implant was still integrated in the mouth, this was considered as positive implant survival. One calibrated investigator performed all the measurements. All the patients were examined clinically and radiographically on the date of implant placement and after 8 years, and a measurement method analogous to a previously published one was used [16]. There are a variety of connections between the implant and the crown [18]. In this study, one-piece zirconia implants were evaluated in which the crown was cemented.

The success rate according to Albrektsson et al. (1986) was used [19]. These researchers postulated a combination of anamnestic data, clinical examination results, and radiological findings for the success rates of implants: no pain or discomfort, immobility, absence of radiolucency, and bone resorption of less than 0.2 mm per year from the timepoint of implant loading.

At the examination after 8 years, the peri-implant clinical parameters were assessed [20–22]. One experienced clinician recorded all the measurements using a plastic probe with a standardized probing force of 0.2 N. The pocket depth was measured at four points around each implant using the mean value for statistical analyses. Additionally, the modified plaque index was measured on all 4 surfaces (buccal, lingual/palatal, mesial, distal) around the implants (scale: 0 = no plaque; 1 = plaque not visible but verified with a probe; 2 = visible plaque; and 3 = massive plaque). The modified sulcus bleeding index was measured around the implants with the following scores: 0 = no bleeding; 1 = isolated bleeding; 2 = confluent linear bleeding; and 3 = severe bleeding.

For the evaluation of bone changes, digital panoramic radiographs (Sirona, Bensheim, Germany) were performed. The defined distance of the individual implant lengths was used to calibrate the radiography pictures. For such calibration purposes in radiographs, cylindrical implants with uniform and quality-regulated production dimensions are excellent. Furthermore, possible magnification during picture collection had no discernible effect. The bone levels were measured from the first bone to the implant contact to the implant shoulder, and from the bone contact of the implant over the papilla tip to the contact point of the crowns.

Additionally, photographs were taken at a 90° angle around each crown. The camera setup was the same at all times (Nikon D3S, 105 mm objective, Tokyo, Japan). For calibration, the length of one crown was measured. All the distances were measured using the ImageJ software (ImageJ, Version 1.52 [23]). The height of the papilla was measured from the contact point of the crowns to the tangent formed at the level of the facial mucosa curvature. The ratio of the papilla height to the crown length was calculated according to Chu et al. [24]. The papilla deficit was measured in millimeters.

To assess the peri-implant soft tissue, the pink esthetic score (PES) according to Fürhauser et al. [25] was used. As in these researchers’ investigations, the photographs were evaluated according to the following seven parameters: mesial and distal papilla, soft-tissue level and contour, alveolar deficit, and soft-tissue color, and texture. The soft tissue of the contralateral tooth served as a reference.

### Statistical analyses

Analyses were performed using the Prism 9 software for Mac OS X (GraphPad, La Jolla, CA) running on Apple OS X. The Kolmogorov–Smirnov normality test was used to check whether the variables were normally distributed. A mixed-effects model was used to identify the differences between immediate and delayed implants.

## Results

A total of 39 patients and 67 zirconia implants (19 immediate, 48 delayed) were included in the present study. The mean age of the patients was 58.8 years (range: 29–84 years). The mean follow-up time for all the cases was 8 years after crown placement. ZrO_2_ monotype implants (Straumann® PURE ceramic implant, Institut Straumann AG, Basel, Switzerland) were investigated. All the implants were inserted using a mucoperiostal flap during surgery, and in all cases, simultaneous autologous augmentation was performed (using autologous bone particles). The definite crown placement was carried out after a 3-month healing period in all the groups; however, in the immediate cases during surgery, a temporary chair-side crown without occlusal contact points was used (Fig. 1A, B). For the delayed cases, a transgingival healing cap was inserted.

The overall survival rate was 100%, and the overall success rate was 89.6%. Around the immediate zirconia implants, the success rate was 94.7%, and around the delayed implants, 87.5%.

**Fig. 2:**
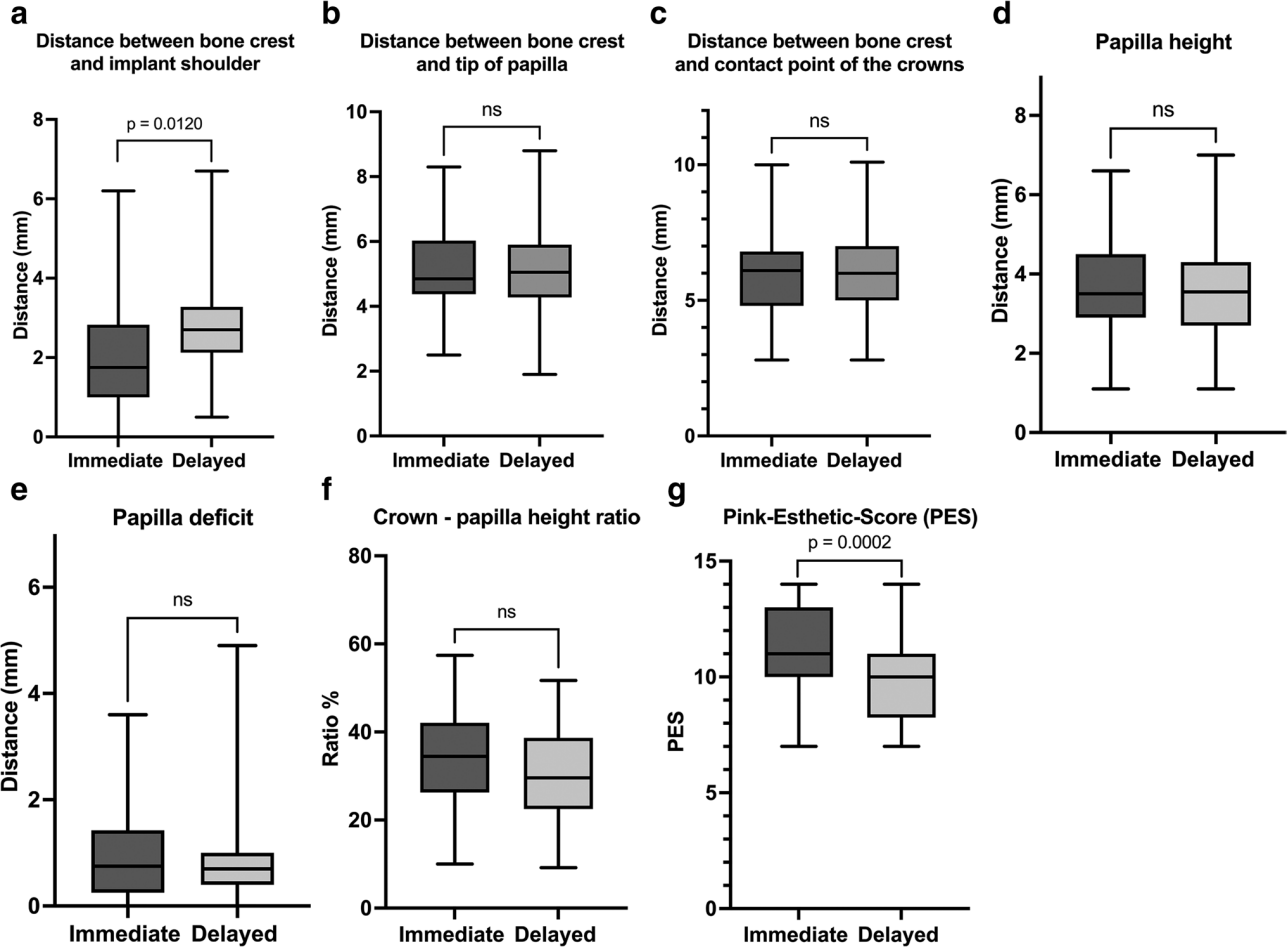
**A** Distance between the bone crest and the implant shoulder (mm). **B** Distance between the bone crest and the papilla tip (mm). **C** Distance between the bone crest and the contact point of the crowns. **D** Papilla height on both sides of the implant. **E** Missing interdental tissue as papilla deficit. **F** Ratio of papilla height to crown length (according to Chu et al. [24]). **G** Pink esthetic score (according to Fürhauser et al. [25]) for assessing peri-implant soft tissue

Regarding the distance between the bone crest and the implant shoulder, the immediate implants showed a significantly shorter distance (e.g., less bone loss) than the delayed implants (Fig. 2A; *p* = 0.0120). On the other hand, no significant differences in the distance between the bone crest and the papilla tip/contact point of the crowns and papilla height (mm) were found between the groups (Fig. 2B–D; *p* > 0.05). In addition, no detectable difference in the papilla deficit and the ratio of the papilla height to the crown length (%) was found between the immediate and delayed zirconia implants (Fig. 2E, F). According to the PES value, the immediate implants had more favorable esthetic results than the delayed implants (11.1 vs. 9.9) after an 8-year follow-up (Fig. 2G, *p* = 0.0002; Table 1).

**Table 1:** Descriptive statistics of measured distances

	Implant	Mean	Standard deviation	Minimum	Maximum
Distance between the bone crest and the implant shoulder (mm)	Immediate	2	1.3	0	6.2
	Delayed	2.8	1.1	0.5	6.7
Distance between the bone crest and the papilla tip (mm)	Immediate	5.1	1.3	2.5	8.3
	Delayed	5.1	1.3	1.9	8.8
Distance between the bone crest and the contact point of the crowns (mm)	Immediate	6.1	1.8	2.8	10
	Delayed	6.1	1.5	2.8	10.1
Papilla height (mm)	Immediate	3.7	1.2	1.1	6.6
	Delayed	3.6	1.2	1.1	7.0
Papilla deficit (mm)	Immediate	1.0	0.9	0	3.6
	Delayed	0.9	0.9	0	4.9
Chu et al. [23] ratio (%)	Immediate	34	10.5	10	57.4
	Delayed	30.4	9.9	9.2	51.7
Pink esthetic score (PES) according to Fürhauser et al. [24] (score)	Immediate	11.1	1.8	7	14
	Delayed	9.9	1.8	7	14
Bone height variations between the bone crest and the implant shoulder up to 8-years (mm)	Immediate	0.4	1.0	−0.7	5.4
	Delayed	0.3	0.6	−0.3	3.0

## Discussion

One-piece zirconia dental implants are characterized by high biocompatibility, low plaque adhesion, and absence of a micro-gap, which can be related to their clinical success [26]. In the present study, we evaluated the survival and success rates of immediate and delayed zirconia implants after an 8-year follow-up. The survival and success rates of immediate zirconia implants were both 100%, while those of delayed zirconia implants were both 89.6%.

In a prospective study, however, only 2 years after implantation, Payer et al. [27] found 95% success and survival rates for monotype zirconia implants. In 2018, Bormann et al. [28] reported similar results for monotype zirconia implants (97.5% survival and success rates after 3 years) from another prospective survey [28]. A 2020 prospective study by Kohal et al. reported a 91.7% 5-year success rate, with a 94.3% survival rate, for both single- and multi-unit zirconia implants [29]. Similar positive results were obtained by Lorenz et al. [30] for 83 monotype implants after a 7.8-year follow-up. Lorenz et al. reported a 100% survival rate; the success rate was not explicitly mentioned, but very good long-term results were reported [30].

According to Francisco et al., both the immediate and delayed implantation methods resulted in an esthetic outcome; no differences in PES were observed [31]. Canellas et al. [32] confirmed the advantage of immediate implant placement with regard to the PES, especially in the anterior region; this may be due to the stable hard- and soft-tissue conditions of the alveoli in the anterior region [32]. The results of our study possibly confirm this thesis as numerous anterior implants (54) were present. Overall, immediate implant placement showed significantly better results for PES than delayed implant placement (*p* = 0.0002). Esposito et al. also reported better esthetic results after immediate implant placement, but they cautioned that higher implant loss rates should be expected in this case [33]. Nevertheless, zirconia implants should be compared to 10 years plus follow-up results of titanium implants. Wennerberg et al. displayed failure percentages of titanium implants of only between 1.6 and 3.3% [34]. In the case of titanium implants, many studies of 10 to 30 years of follow-up are available. Zirconia implants are behind so far, and further long-term studies are necessary.

The aforementioned short- to medium-term studies reported clinically equivalent results for immediate versus delayed implant placement [35, 36]. However, long-term data are scarce; in their 10-year study, Schropp et al. [37] found equally good survival rates after immediate and delayed implantation with titanium (93% and 100%, respectively); no significant differences were found with regard to the distance from the bone at the implant to the implant shoulder [37]. In the present 8-year study with zirconia, however, a significantly shorter distance was found after immediate implant placement than after delayed implant placement.

Our survey revealed that the immediate implant placement had no visual disadvantages and no significant functional limitations in the long term; it had a high survival rate and low bone resorption. However, it must be noted that in our patients, the indication for immediate implant placement was given by an experienced surgeon. These indications included the absence of inflammation, good bone volume, no mucosal disease, no untreated periodontitis, or gingivitis, no severe bruxism or clenching habits, and a compliant patient (no hard food contact to the immediate implant crown) during the 3-month healing period. It was observed that delayed implants had lower levels of success. Delayed implants were preferred in certain compromised bone situations. This may be the reason for the reduced success rate.

According to the criterion of Tarnow et al. [38] and Choquet et al. [39] (≤ 5 mm distance between the bone crest and the papilla tip), full papilla formation would have been achieved in only about half of the cases in the present study as the mean distance from the bone attachment at the implant to the papilla tip was 6.1 mm.

Chu et al. found an average quotient of about 40% and defined this as the ideal esthetic value. However, they pointed out that there was still a subjective range of variation and that an esthetic ideal could be subject to social and cultural influences [24]. The data in the present study yielded 31.8% and 31.4% median and mean papilla height-to-crown length ratios, respectively. Thus, only a few of the ratios obtained reached the ideal postulated by Chu et al. [24].

A slightly increased risk of implant loss 1 and 5 years after immediate titanium implantation, respectively, was reported in Canellas et al.’s ^32^ and Esposito et al.’s ^33^ meta-analyses. In contrast, in our long-term study exclusively related to zirconia, a 100% survival rate was observed after immediate implantation.

With the modern zirconia two-part variants, the crown can be placed via angulation. Tilted implants can be an effective and safe alternative to avoid augmentation procedures [40].

In addition to the clinical survey, all the implants were examined radiographically. Thus, possible periapical abnormalities or radiolucencies could be excluded (no implant in the present study was conspicuous), and functionally relevant distances could be determined.

In the present study, each radiographic examination was performed using panoramic radiography, a widely used method for clearly and reproducibly visualizing the entire jaw with a low radiation dose [41]. Chopra et al. also described it as suitable for evaluating the bone site before and after implant placement, especially with regard to osseointegration after insertion [42]. Another advantage of the method is its manageability and low cost [43]. Further future studies around zirconia implants are necessary. The success rate in diabetic patients should also be investigated [44].

## Conclusison

After 8 years, one-piece zirconia implants presented an 89.6% success rate. Around the immediate zirconia implants, a higher bone crest was found, and the esthetic results were more favorable than those of the delayed implants.

## Data Availability

Due to the sensitive nature of the data, information created during and/or analyzed during the current study is available from the corresponding author (Kristian Kniha) on reasonable request.
